# Menstrual Blood-Derived Stromal Stem Cells Augment CD4+ T Cells Proliferation

**Published:** 2018

**Authors:** Mehdi Aleahmad, Alireza Ghanavatinejad, Mahmood Bozorgmehr, Mohammad-Reza Shokri, Shohreh Nikoo, Maryam Tavakoli, Somaieh Kazemnejad, Fazel Shokri, Amir-Hassan Zarnani

**Affiliations:** 1. Department of Immunology, Faculty of Public Health, Tehran University of Medical Sciences, Tehran, Iran; 2. Reproductive Immunology Research Center, Avicenna Research Institute, ACECR, Tehran, Iran; 3. Oncopathology Research Center, Iran University of Medical Sciences, Tehran, Iran; 4. Department of Immunology, Faculty of Medicine, Iran University of Medical Sciences, Tehran, Iran; 5. Immunology Research Center (IRC), Iran University of Medical Sciences, Tehran, Iran; 6. Reproductive Biotechnology Research Centre, Avicenna Research Institute, ACECR, Tehran, Iran; 7. Department of Hybridoma, Monoclonal Antibody Research Center, Avicenna Research Institute, ACECR, Tehran, Iran

**Keywords:** Endometrium, Immunological tolerance, Menstrual blood stem cells, Pregnancy, Proliferation, T lymphocytes

## Abstract

**Background:**

It is more than sixty years that the concept of the fetal allograft and immunological paradox of pregnancy was proposed and in this context, several regulatory networks and mechanisms have been introduced so far. It is now generally recognized that mesenchymal stem cells exert potent immunoregulatory activity. In this study, for the first time, the potential impact of Menstrual blood Stem Cells (MenSCs), as surrogate for endometrial stem cells, on proliferative capacity of CD4+ T cells was tested.

**Methods:**

MenSCs and Bone marrow Mesenchymal Stem Cells (BMSCs) were isolated and assessed for their immunophenotypic features and multi-lineage differentiation capability. BMSCs and MenSCs with or without IFNγ pre-stimulation were co-cultured with purified anti-CD3/CD28-activated CD4+ T cells and the extent of T cell proliferation at different MenSCs: T cell ratios were investigated by CSFE flow cytometry. IDO activity of both cell types was measured after stimulation with IFNγ by a colorimetric assay.

**Results:**

MenSCs exhibited dual mesenchymal and embryonic markers and multi-lineage differentiation capacity. MenSCs significantly increased proliferation of CD4+ cells at ratios 1:2, 1:4 and 1:8. IFNγ pre-treated BMSCs but not MenSCs significantly suppressed CD4+ T cells proliferation. Such proliferation promoting capacity of MenSCs was not correlated with IDO activity as these cells showed the high IDO activity following IFNγ treatment.

**Conclusion:**

Although augmentation of T cell proliferation by MenSCs can be a basis for maintenance of endometrial homeostasis to cope with ascending infections, this may not fulfill the requirement for immunological tolerance to a semi-allogeneic fetus. However, more investigation is needed to examine whether or not the immunomodulatory properties of these cells are affected by endometrial microenvironment during pregnancy.

## Introduction

One of the most controversial issues in reproductive biology is dealing with the fact that a fully functional immune system in women should simultaneously fight off the invading pathogens and tolerate semi-allograft fetus throughout the pregnancy. Indeed, a successful pregnancy is supposed to remain unresponsive to paternal antigens originating from semi-allograft fetus.

Thus far, extensive attempts and studies have been performed to unravel immunosuppressive mechanisms involved in immunological tolerance of gestation. Endometrium undergoes immunological changes to establish tolerance during the onset of pregnancy. Along with gestation initiation, such immune cells as Natural Killer cells (NKs), monocytes, Dendritic Cells (DCs) and T cells are recruited to the endometrium. The phenotype of decidual immune cells changes in a way to cooperate with tolerance. Recruited NK cells, for instance, transform into decidual NK cells (dNK) with a reduced cytotoxic and augmented secretary activity 1–3. Macrophage and NK cells together induce tolerogenic DCs (tDCs) 4, which per se promote Treg differentiation. Nevertheless, it has been reported that depleting Tregs causes only a 10% fetal loss in the first pregnancy of mice 5. Indeed, there is evidence that Fas (First apoptosis signal), Indoleamine 2,3-dioxygenase (IDO) and Programmed Death-Ligand 1 (PD-L1) suppress fetus antigen-specific effector T cells 6–8, but immunotolerance is not interrupted even if one of these factors is absent in allogeneic matings in *Ido1*−*/* − or *Fasl*−*/* − mice 6,9. Although redundancy and overlapping compensatory mechanisms may explain in part the aforesaid phenomenon, one tempting hypothesis would be immunomodulation at the feto-maternal interface by non-immune cells residing in the endometrium.

Immunomodulatory functions are not limited to immune cells. Numerous researches have addressed immunomodulation as the prominent feature of Mesenchymal Stem Cells (MSCs). Plenty of studies have shown that MSCs derived from a variety of tissues such as bone marrow, adipose and amniotic membrane have immunomodulatory properties exemplified by suppressing T cell activation and proliferation 10–14.

In 2004, the existence of a specific population of cells in the endometrium with ability to form Colony Forming Unit (CFU) was introduced 15,16. Subsequently, it was reported that CD146+ colonogenic human perivascular endometrial stromal cells might be potential stromal stem/progenitor cells 17. Complementary information was provided by Gargett *et al* who showed that endometrial colonogenic stromal cells possess all criteria that a cell needs to be categorized as MSCs 18. Based on non-invasive method of collection, menstrual blood as a source for a MSCs originated from endometrium was then extensively investigated. It was observed that menstrual blood-derived stem cells contained heterogeneous cell populations, expressed MSCs markers and were able to differentiate into chondrogenic, adipogenic, and osteogenic cell lineages 19. In addition, they observed a similarity between endometrial and Menstrual Blood Stem Cells (MenSCs) with respect to the expression of c-Kit 20 and Oct-4 21; they concluded that MenSCs are possibly endometrium MSCs shed during menstruation 19.

Although more than a decade since the first introduction of endometrial stem cells in general and the menstrual blood stem cells, in particular, have passed, there is very limited data on their potential immunoregulatory capacity. Previously, our group demonstrated that MenSCs dampen allogeneic MLR 22 and interfere with the process of DC differentiation and maturation 23.

Given the presence of T cells in endometrium and their pivotal role in maintenance of successful pregnancy and also in pregnancy related complications such as abortion, in this study, an attempt was made to explore how endometrial mesenchymal stromal cells control CD4+ T cells responses.

## Materials and Methods

### MenSCs and BMSCs collections

MenSCs were obtained from 10 apparently healthy women (25–35 years). The women were monitored to exclude those with a history of vaginal infection or consumption of oral contraceptives, corticosteroids and Nonsteroidal Anti-inflammatory Drug (NSAIDs) during the last 3 months, endometriosis, autoimmune diseases and infection with such blood transmittable viruses such as HCV, HBV and HIV. A written consent was obtained from all donors before enrolment to the study. BMSCs were from four healthy donors admitted for bone marrow transplantation and provided by Reproductive Biotechnology Research Center, Avicenna Research Institute, Tehran, Iran. MenSCs were collected on the 2^nd^ day of menstruation phase using menstrual cup. Samples were transferred to the lab in a transfer medium comprising DMEM/F12, 100 *μg/ml* penicillin, 100 *IU/ml* streptomycin and 0.25 *μg/ml* fungizone (Invitrogen, Carlsbad, CA). Clots and tissue derbies were separated using cell strainer with 70 *μm* pore size. Then, menstrual blood was cultured in DMEM/F12 media supplemented with 10% Fetal Bovine Serum (FBS) (Invitrogen, Carlsbad, CA) and with the same concentration of antibiotics as mentioned above. Every two or three days, media were replenished, suspended cells were removed and the adherent cells were passaged up to 5 times. These cells were considered as MenSCs and frozen for the following experiments.

### Immunophenotyping

MenSCs and BMSCs were harvested after two passages and evaluated for their immunophenotype characteristics using antibodies against MSCs markers: CD9, CD10, CD44, CD73 and CD105, embryonic stem cells markers; Oct-4, Nanog, Stro-1 and SSEA-4 and hematopoietic markers; CD34, CD38, CD45, and CD133. The specification of antibodies is summarized in table 1.

**Table 1.** Antibody panel for immunophenotyping

**Antibody**	Fluorochrome	Clone	Company
**Anti-CD9**	FITC	M-L13	BD Bioscience
**Anti-CD10**	PE	HI10a	BD Bioscience
**Anti-CD29**	PE	MAR4	BD Bioscience
**Anti-CD34**	FITC	581	BD Bioscience
**Anti-CD38**	FITC	HIT2	BD Bioscience
**Anti-CD44**	PE	515	BD Bioscience
**Anti-CD45**	PE	HI30	BD Bioscience
**Anti-CD73**	PE	AD2	BD Bioscience
**Anti-CD105**	PE	166707	R&D systems
**Anti-CD133**	PE	W6B3C1	BD Bioscience
**Anti-Nanog**	-	Polyclonal	Abcam
**Anti-Oct-4**	-	Polyclonal	Abcam
**Anti-SSEA-4**	-	MC813-70	BD Bioscience
**Anti-Stro-1**	-	STRO-1	R&D systems
**Anti-rabbit Ig**	FITC	Polyclonal	Abcam
**Anti-mouse IgG**	FITC	Polyclonal	Sina Biotech

### Multi-lineage differentiation

MenSCs and BMSCs were differentiated into adipogenic, chondrogenic and osteogenic lineages using specific polarizing media as per method described previously 24,25. In brief, MenSCs and BMSCs were seeded in 24-well plates at 5×10^4^ cell/well. For adipogenic differentiation, MenSCs or BMSCs were cultured in DMEM-F12/FBS 10% supplemented with 1 *μM* rosiglitazone (St Louis, MO, USA), 10 *μg/ml* human recombinant insulin, 0.5 *mM* IBMX (3-Isobutyl-1-methylxanthine) (St Louis, MO, USA) and 1 *μM* dexamethasone (Cosar Pharmaceutical Company). Chondrogenic differentiation medium was made of DMEM-F12/FBS 10% comprising 100 *μg/ml* sodium pyruvate (Invitrogen, Carlsbad, CA), 20 *ng/ml* TGF-β3 (St Louis, MO, USA), 100 *nM* dexamethasone, ITS+1 1X (St Louis, MO, USA), 50 *μg/ml* ascorbic acid (St Louis, MO, USA) and 2% FBS. To differentiate into osteogenic lineage, culture media contained complete high glucose DMEM supplemented with 0.1 *μM* dexamethasone, 50 *μM* ascorbic acid and 10 *μM* β-glycerophosphate (Sigma, St Louis, MO, USA). As control wells, the same cell number was seeded in the same plates without any polarizing agents. To evaluate differentiation into adipogenic, chondrogenic and osteogenic lineages, Oil red, Alcian blue and Alizarin red staining was employed, respectively.

### T cell isolation and co-culture

Peripheral blood samples were obtained from healthy donors. Then Peripheral Blood Mononuclear Cells (PBMCs) were isolated using density gradient Ficoll paque medium (Amersham, UK). CD4+ T cells were purified from PBMCs using magnetic beads negative selection kit (Miltenyi Biotec, Germany) with approximate purity of 95%. CD4+ T cells were co-cultured (at 4×10^5^ cells/well) with MenSCs at 1:2 to 1:128 ratios (MenSCs: CD4+ T cells) in 24-well plates for five days. During culture, CD4+ T cells were stimulated with anti-CD3 and anti-CD28-loaded activation beads at a ratio of 1:4 (bead:cell) (Miltenyi Biotec, Germany).

### Pre-treatment of MenSCs with IFNγ

In some settings, MenSCs and BMSCs were stimulated with 25 *ng/ml* IFNγ in 24-well plates for 48 *hr* before co-culture with CD+ T cells. Thereafter, Men-SCs and BMSCs were co-cultured for five days with CD4+ T cells as above, at ratios of (MSCs: CD4+ T cells) 1:4–1:8 and 1:5, respectively.

### Proliferation assay

The modulatory action of MSCs on T cells proliferation was investigated by CFSE flow cytometry. To this end, CFSE-labeled (Molecular probe, USA) CD4+ T cells were cultured in the presence or absence of MSCs for five days, harvested and analyzed using flow cytometry (Attune NXT, Thermo Fisher, Carlsbad, USA). For CFSE labeling, CD4+ T cells were stained with 5 *μM* CFSE dye solution and washed two times prior to co-culture with MSCs.

### IDO activity assay

IDO activity in MenSCs and BMSCs supernatant was assessed with or without IFNγ pre-stimulation. MenSCs or BMSCs were seeded at 1×10^5^ cell/well in 650 *μL* DMEM-F12/FBS 10% (24-well plate). To evaluate IDO activity, 100 *μg/ml* tryptophan (Sigma, St Louis, MO, USA) was added to each well in the presence or absence of 100 *ng/ml* IFNγ (control wells contained only culture media) and incubated in a humidified incubator for 48 *hr*. Supernatant was harvested and prepared as described elsewhere 26. IDO activity was then assessed through measurement of tryptophan catabolite (kynurenine) concentration, using a plate reader (Biotec, VT, USA) at 450 *nm*.

### Statistical analysis

Flow cytometry data were analyzed using Flowjo 7.6.1 Software (Tree Star Inc., Ashland, USA). All colorimetric experiments were performed in triplicate. Mann-Whitney was used to evaluate the differences. All graphs are displayed using median and rage. P values less than 0.05 were considered statistically significant. The analysis was done using Prism software 6.0 (GraphPad Software Inc., San Diego, USA).

## Results

### MenSCs exhibited dual mesenchymal and embryonic markers and multi-lineage differentiation capacity

**Figure 1.**
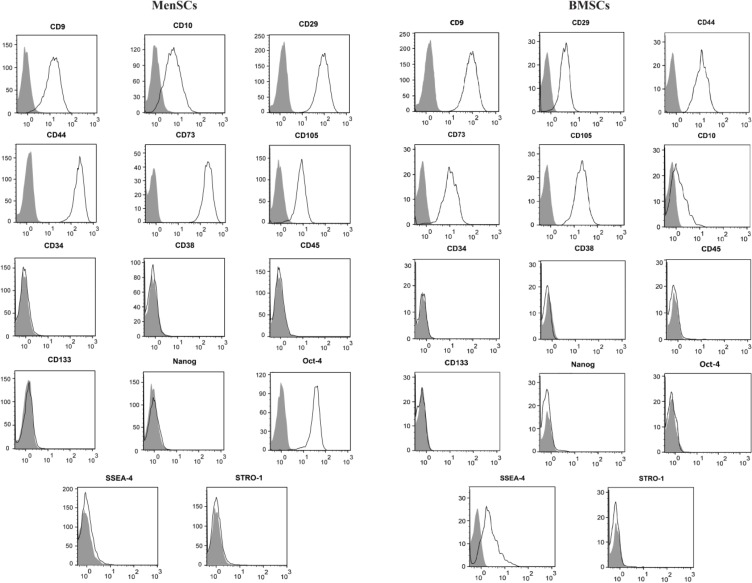
Immunophenotyping of MenSCs and BMSCs. MenSCs and BMSCs were evaluated for the expression of MSCs markers, CD9, CD10, CD29, CD44, CD73 and CD105, hematopoietic makers, CD34, CD38, CD45 and CD133, and pluripotency makers, Nanog, Oct-4, SSEA-4 and Stro-1. The grey and empty histograms represent unstained sample and test samples, respectively. Results are representative of three individual experiments.

**Figure 2.**
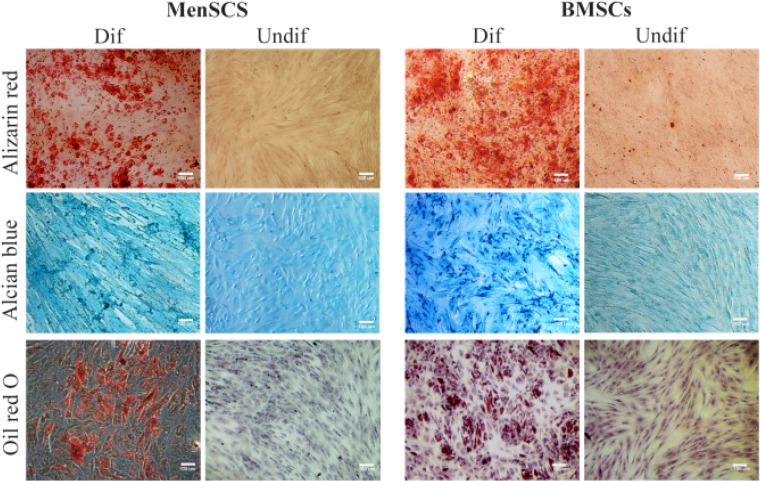
Multi-lineage differentiation potential of MenSCs and BMSCs. The left and right pictures of each panel represent differentiated (Dif) and undifferentiated (Undif) stem cells, respectively. Differentiation of stem cells toward osteocytes, chondrocytes and adipocytes were assessed by Alizarin red, Alcian blue and Oil red staining, respectively. Results are representative of three individual experiments.

MenSCs and BMSCs expressed MSCs markers including CD9, CD10, CD29, CD44, CD73 and CD105, they were also negative for hematopoietic markers, CD34, CD38, CD45 and CD133. MenSCs also expressed Oct-4 but failed to express SSEA-4, while the opposite pattern was the case for BMSCs (Figure 1), (Table 2). Both MenSCs and BMSCs were capable of differentiating into adipogenic, chondrogenic and osteogenic lineages confirming their MSCs identity. Men-SCs showed less potency to differentiate into osteogenic and adipogenic lineages compared to BMSCs (Figure 2).

**Table 2.** Expression of mesenchymal and embryonic stem cell markers by MenSCs and BMSCs

**Markers**	**MenSCs**	**BMSCs**
**CD34**	1.7±0.9%	0.92±0.4%
**CD38**	1.4±0.7%	1.9±1.3%
**CD45**	1.1±0.7%	1.7±0.8%
**CD133**	1.9±0.7%	2.1±1.6%
**CD9**	90.8±6.7%	92.1±5%
**CD10**	89.4±5.7%	40.1±18%
**CD29**	98.7±1.26%	94.9±5%
**CD44**	99±0.2%	96.97±3%
**CD73**	99.7±0.2%	97.6±1.2%
**CD105**	89.6±9.6%	99.7±0%
**Stro-1**	4.7±1.4%	8.7±3.4%
**Oct-4**	99.5±0%	1.4±1.1%
**Nanog**	2.5±0.4%	7±2.4%
**SSEA-4**	1.8±0.8%	78.3±11.4%

### MenSCs augmented CD4+ T cells proliferation

**Figure 3.**
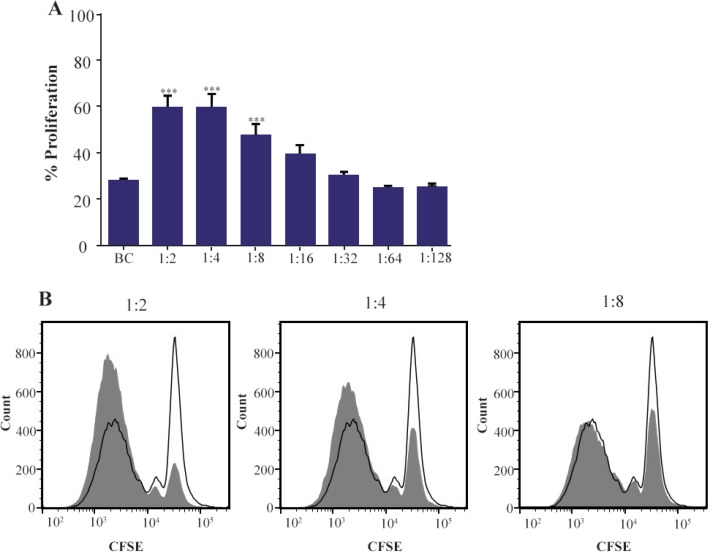
Effect of MenSCs on proliferation of CD4+ T cells. A) MenSCs were co-cultured at different ratios with anti-CD3/CD28-activated purified CD4+ T cells for 5 days and the percent of proliferation was assessed by CFSE flow cytometry. B) Representative histogram plots are shown. The grey and empty histograms represent test samples (co-culture) and biological controls (BC) (CD4+ T cells cultured alone), respectively. Results are representative of nine individual experiments. ***: p<0.001.

To assess the immunomodulatory capability of Men-SCs, they were co-cultured with CFSE-labeled anti-CD3/anti-CD28-activated CD4+ T cells at different ratios. As shown in figure 3, MenSCs significantly increased proliferation of CD4+ cells at ratios 1:2, 1:4 and 1:8 (p<0.001). Although the rate of proliferation at higher ratios was higher compared to the CD4+ T cells cultured alone, the differences were not reached to the statistically significant level.

### IFNγ pre-treated BMSCs but not MenSCs suppressed CD4+ T cells proliferation

**Figure 4.**
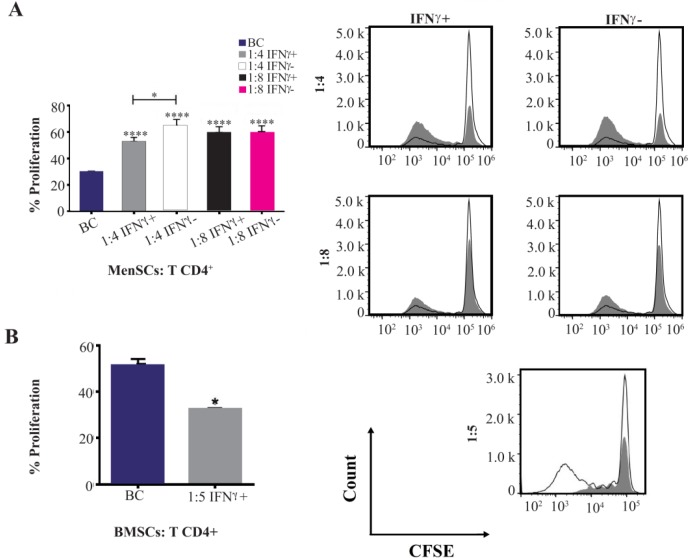
Effect of IFNγ stimulation of MenSCs on proliferation of CD4+ T cells: A) MenSCs were co-cultured with CD4+ T cells at 1:4 and 1:8 (MenSCs:CD4+ T cells) ratios with or without IFNγ pre-stimulation for five days and the percent of proliferation was assessed by CFSE flow cytometry. B) IFNγ pre-stimulated BMSCs were used as positive control in CD4+ T cells proliferation assay. Figures on the right in each panel represent histogram plots of corresponding proliferation assays. The empty histograms represent biological controls (BC) (CD4+ T cells cultured alone) and grey histograms represent test samples (co-culture). Results are representative of ten individual experiments *: p<0.05 and ****: p<0.0001.

BMSCs as the most studied source of MSCs with potent immunomodulatory impact on T cell proliferation upon stimulation with pro-inflammatory cytokines such as IFNγ, IL-1β and TNF-α were tested as positive control. IFNγ pre-stimulated BMSCs significantly suppressed proliferation of CD4+ T cells compared to the control wells (p<0.05). Although CD4+ T cells proliferation was reduced in the presence of IFNγ pre-stimulated MenSCs (p<0.05), it was still significantly higher than the control (p<0.0001) (Figure 4).

### IFNγ induced IDO activity in both MenSCs and BMSCs

**Figure 5.**
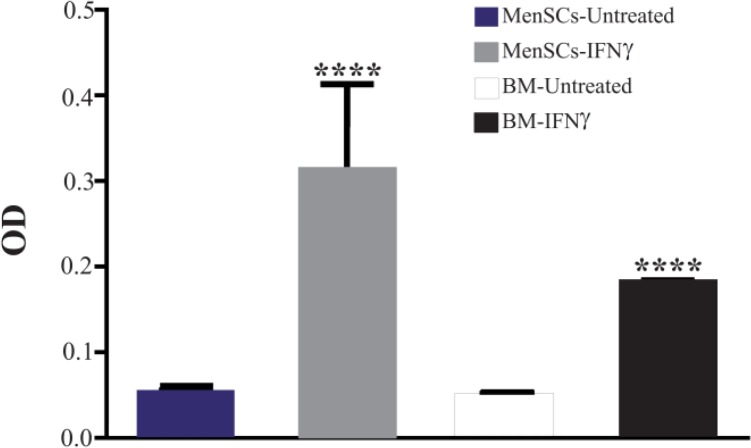
Assessment of IDO activity in MenSC and BMSC supernatants after stimulation with IFNγ. IDO activity was measured using kynurenine colorimetric assay. The results are median and rage of four BMSCs and six MenSCs samples ****: p<0.0001.

IDO has been widely studied due to its role in tolerance. IFNγ is the most potent stimulator of IDO activity. In this context, MenSCs and BMSCs were stimulated with IFNγ and IDO activity was measured in cell culture supernatant. Our results showed that in Men-SCs, IDO activity was induced in both cell types after stimulation with IFNγ compared to the un-treated cells. Both MenSCs and BMSCs exhibited higher IDO activity compared with controls, after stimulation with IFNγ (p<0.0001) (Figure 5).

## Discussion

Although plenty of mechanisms and regulatory networks for establishment of immune tolerance at the feto-maternal interface have been introduced, the potential immunomodulatory role of endometrial stromal stem cell has been largely ignored. During the past couple of years, the immunomodulatory properties of mesenchymal stem cells have attracted interest of many researchers and to a large extend have foregrounded the principal application of this cell population in regenerative medicine. In this study, the potential immunomodulatory impact of MenSCs, as surrogate cells for endometrial mesenchymal stem cells, on T cell proliferation was addressed. As with previous reports 25, it was shown that MenSCs possessed minimal criteria necessary for defining a cell type as MSCs exemplified by the expression of markers associated with mesenchymal origin and multi-lineage differentiation 25. Expression of the embryonic marker, Oct-4, by MenSCs is a further support to the previous reports on higher proliferation capacity of these cells compared to BMSCs 27.

In the next step, the potential modulatory effect of MenSCs on CD4+ T cells proliferation was examined in reference to BMSCs. It was shown that at MenSCs: T cell ratios of 1:2–1:8, MenSCs supported CD4+ T cells proliferation. This finding seems to have contradiction with our previous results 22, because in that report MenSCs were able to suppress allogenic MLR at 1:1 and 1:2 (MenSCs: PBMCs) ratios. Notably, in allogeneic MLR, a mixture of pro-inflammatory cytokines profiles is produced by responder cells including IL-1β and TNF-α 28 which are able to induce anti-inflammatory phenotype in MSCs 29. Hence, it could be inferred that inflammatory milieu during MLR reaction may help to boost MSCs immunomodulatory capabilities. On the other hand, MSCs use monocyte-dependent mechanism to halt T cell responses 30–32 which was absent in the system reported here. Although by taking our initial concept into consideration, this finding was out of our expectation, the following explanation could be put forth. The upper part of female reproductive tract is sterile and in non-pregnant women, immune system needs to be on a stand-by mode to properly respond to any invading pathogen; hence, it seems logical to assume that every potential immunotolerance mechanism remains at low functional level during non-pregnant state 33. Dual anti-inflammatory or pro-inflammatory phenotype of MSCs depending upon microenvironment milieu has already been reported 34.

The onset of pregnancy and blastocyst implantation is associated with inflammatory processes initiated by insemination 35 and recruitment of dNK cells. Besides endometrial immune cells such as dNK cells which produce IFNγ 36,37, endometrial non-immune cells are also a potential source for establishment of inflammatory milieu 38. Interestingly, most MSCs acquire anti-inflammatory phenotype upon treatment with such pro-inflammatory cytokines as IFNγ 39–41. With this in mind, effect of IFNγ pre-treatment on modulatory activity of MenSCs on the proliferative response of CD+ T cells was evaluated in the next step.

As expected, IFNγ-treated BMSCs significantly inhibited T cell proliferation, which was in accordance with results reported by other groups 10,42,43. Although IFNγ treatment of MenSCs reduced their capacity to augment T cell proliferation, it was still significantly higher than control. This finding may be due to the lower expression level of IFNγ receptor in MenSCs compared with BMSCs 44. Almost a similar result was observed in umbilical cord-derived MSCs co-cultured with PHA-activated PBMCs 45. On the other hand, induction of IDO activity in MenSCs treated with IFNγ implies that suppressive activity of MenSCs IDO on T cell proliferation was not sufficient enough to overcome yet undetermined proliferation supportive mechanisms of this cell population.

It is notable that, IFNγ is not the only pro-inflammatory cytokine in early pregnancy decidua. Expression of other pro-inflammatory cytokines including IL-1, TNF-α, and IL-18 in early pregnancy decidua is up-regulated 46. Interestingly, IL-1β and TNF-α are among the pro-inflammatory cytokines that have been proven to induce anti-inflammatory phenotype in MSCs 47–51. Thus, it remains to be investigated whether endometrial microenvironment during pregnancy can affect the immunomodulatory properties of MenSCs.

## Conclusion

Our results showed that MenSCs induce proliferation of CD4+ T cells which could be a basis for maintenance of endometrial homeostasis to cope with ascending infections. This feature, however, seems to be contradictory to the requirement for immunological tolerance to semi-allogeneic fetus. Whether or not this immune enhancement capacity of MenSCs is modulated during pregnancy under the influence of immunosuppressive hormones and mediators needs to be determined.
